# Structure and Mechanism of the Bifunctional CinA Enzyme from *Thermus thermophilus* *

**DOI:** 10.1074/jbc.M114.608448

**Published:** 2014-10-13

**Authors:** Vijaykumar Karuppiah, Angela Thistlethwaite, Rana Dajani, Jim Warwicker, Jeremy P. Derrick

**Affiliations:** From the Faculty of Life Sciences, University of Manchester, Michael Smith Building, Oxford Road, Manchester M13 9PT, United Kingdom

**Keywords:** DNA Transformation, Enzyme Catalysis, Hydrolase, Nicotinamide Adenine Dinucleotide (NAD), X-ray Crystallography, ADP-ribose Pyrophosphatase, COG1058 Domain, Nicotinamide Mononucleotide Deamidase

## Abstract

CinA is a widely distributed protein in Gram-positive and Gram-negative bacteria. It is associated with natural competence and is proposed to have a function as an enzyme participating in the pyridine nucleotide cycle, which recycles products formed by non-redox uses of NAD. Here we report the determination of the crystal structure of CinA from *Thermus thermophilus*, in complex with several ligands. CinA was shown to have both nicotinamide mononucleotide deamidase and ADP-ribose pyrophosphatase activities. The crystal structure shows an unusual asymmetric dimer, with three domains for each chain; the C-terminal domain harbors the nicotinamide mononucleotide deamidase activity, and the structure of a complex with the product nicotinate mononucleotide suggests a mechanism for deamidation. The N-terminal domain belongs to the COG1058 family and is associated with the ADP-ribose pyrophosphatase activity. The asymmetry in the CinA dimer arises from two alternative orientations of the COG1058 domains, only one of which forms a contact with the KH-type domain from the other chain, effectively closing the active site into, we propose, a catalytically competent state. Structures of complexes with Mg^2+^/ADP-ribose, Mg^2+^/ATP, and Mg^2+^/AMP suggest a mechanism for the ADP-ribose pyrophosphatase reaction that involves a rotation of the COG1058 domain dimer as part of the reaction cycle, so that each active site oscillates between open and closed forms, thus promoting catalysis.

## Teaser

**Background:** CinA is a bifunctional enzyme that catalyzes reactions associated with pyridine nucleotide recycling in bacteria.

**Results:** the structure of the enzyme shows an unusual asymmetric dimer with one of the catalytic sites in two different conformational states.

**Conclusion:** Mechanisms for both reactions are proposed, based on structures of ligand complexes.

**Significance:** Asymmetric enzyme dimers are rare; in this case, domain movement is necessary to promote catalysis.

## Introduction

Competence is the name given to the physiological state by which bacteria are able to take up DNA from outside the cell and, through a multistep process, incorporate it into their genome (1). In some organisms, competence is induced through limited nutrient conditions (*e.g.* in *Bacillus subtilis* (2)). *Thermus thermophilus*, on the other hand, is constitutively competent, demonstrating very high rates of natural transformation (2). Uptake of DNA is carried out by a dedicated complex of several proteins that are responsible for the binding of DNA and the transport of single-stranded DNA into the cytoplasm (3). From there, incorporation of single-stranded DNA fragments into the chromosome requires the use of DNA repair proteins, such as RecA (4, 5). In the *T. thermophilus* HB8 genome, the *recA* gene (TTHA1818) is preceded by a predicted 2′–5′ RNA ligase (TTHA1819) and a protein denoted as *cinA* (TTH1820). The nomenclature of *cinA* is derived from its association with competence (competence-induced protein), and *cinA* mutants in streptococci result in reduced transformation efficiency (4–6). In *B. subtilis* and *Streptococcus pneumoniae*, *cinA* and *recA* are adjacent in the genome in a manner suggestive of a related function, and there is evidence that the two proteins from *S. pneumoniae* form a complex (5). A mutagenesis study in *B. subtilis*, however, found little effect of a *cinA* knockout on competence (2). Curiously, sequence analysis suggested a similarity between a domain in CinA and an enzyme involved in molybdopterin biosynthesis, but there was little clear indication how these disparate observations might be linked (2).

**FIGURE 1.**
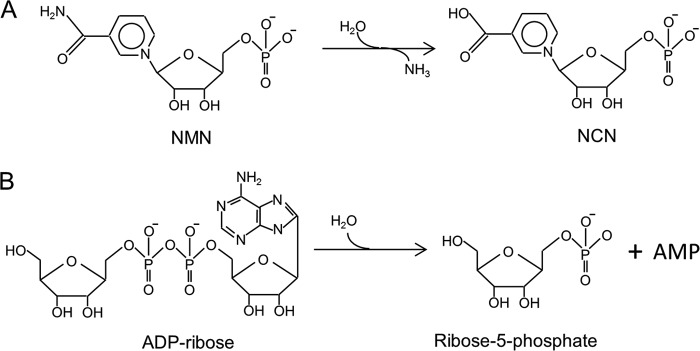
**Enzymatic activities of CinA-like proteins.** *A*, NMN deamidase; *B*, ADPR.

Recent studies on CinA proteins from several bacterial sources have shed more light on this conundrum. Galeazzi *et al.* (7) identified a nicotinamide mononucleotide (NMN)^2^ deamidase activity in a CinA-like protein from *Shewanella oneidensis*. Purification of this protein, termed PncC, directly from *S. oneidensis* verified that it was capable of NMN deamidase activity, which transforms NMN into nicotinate mononucleotide (NCN; Fig. 1*A*). This reaction forms part of the pyridine nucleotide cycle; NCN is subsequently transformed by an adenylyltransferase, NadD, to nicotinate adenine dinucleotide, which is a substrate for NAD synthase (NadE). One function of the pyridine nucleotide cycle is to recycle NAD precursors that are generated from non-redox reactions involving NAD. An example of such a reaction is the NAD-dependent DNA ligase, which transfers the AMP moiety of NAD onto the 5′-phosphate of the DNA substrate (8). The turnover of the NAD pool in bacteria can be rapid; Cheng and Roth (9) estimated that *Salmonella typhimurium* turns over half of its NAD pool every 90 min under aerobic conditions. Furthermore, one product of DNA ligase, NMN, is an inhibitor of the ligase reaction (9). These observations led Galeazzi *et al.* (7) to suggest that increased DNA ligase activity could be associated with an enhanced level of recombination occurring as a result of DNA uptake during competence.

Homologs of the NMN deamidase domain, as identified by Galeazzi *et al.* (7), are found as single domains or, in about 40% of cases (10), as a fusion with a domain that is similar in sequence to a molybdopterin biosynthesis enzyme, MoeA/MocF (11). This observation led Cialabrini *et al.* (11) to investigate the function of this domain, designated as COG1058 in the Clusters of Orthologous Groups database (12) and demonstrate that it has ADP-ribose pyrophosphatase (ADPR) activity.

Sánchez-Carrón *et al.* (10) conducted a phylogenetic analysis of CinA sequences; most sequences that contain the fusion to the COG1058 domain are found in Gram-positive bacteria, but there was also significant representation from Cyanobacteria, Proteobacteria, and Bacteroidetes. To date, two crystal structures of representative domains from CinA-like proteins have been described. Protein Data Bank entry 2A9S from *Agrobacterium tumefaciens* corresponds to the NMN deamidase domain; it forms a dimer with an α/β fold and an extensive monomer-monomer interface. The NMN substrate was modeled into a crevice on the enzyme surface by Galeazzi *et al.* (7) but without independent experimental verification. The second structure, Protein Data Bank entry 3KBQ from *Thermoplasma acidophilum*, corresponds to the COG1058 domain and is therefore different from 2A9S. Sánchez-Carrón *et al.* docked the proposed substrate, ADP-ribose, into the active site (10), but again, this was not verified experimentally. Furthermore, the relationship of these domains in the multiple-domain CinA variants is unclear. Here we describe the determination of the crystal structure of CinA from *T. thermophilus* (TtCinA), which contains both the NMN deamidase and COG1058 domains and is therefore a bifunctional enzyme. We demonstrate that TtCinA adopts an asymmetric dimer and present structures with NCN, ADP-ribose, AMP, and ATP bound, which provide experimental validation for the modes of substrate binding proposed previously (7, 10). In addition, we show that TtCinA adopts a three-domain structure, with a third, previously unidentified, KH-type domain between the NMN deamidase and COG1058 domains. We use these structures to propose models for both enzymatic activities and suggest that large scale movement of the COG1058 domains is linked to ADPR catalysis as well as providing a rationale for the usual asymmetric arrangement of the TtCinA dimer.

## EXPERIMENTAL PROCEDURES

### 

#### 

##### Recombinant Expression and Purification of TtCinA

The *cinA* gene was amplified from *T. thermophilus* H8 genomic DNA using the primer set CTTTAAGGAGGCCATATGGAGCGGGCAGAGATC and GACCGCGTAGAGAATTCTCATGTCACAAGGAG. The amplified gene and the pET28a vector were treated with NdeI and EcoRI restriction enzymes, purified, and ligated. The expression construct, *cinA*-28a, encoded the full-length CinA protein with a His_6_ tag followed by a thrombin cleavage site at the N terminus. The *cinA*-28a plasmid was transformed into T7 Express cells (New England Biolabs), and transformants were inoculated into 50 ml of Terrific Broth medium (ForMedium) containing 40 μg/ml kanamycin. After 3–4 h, the start-up culture was diluted into 2 liters of fresh Terrific Broth medium, and the cells were allowed to grow at 37 °C until the *A*_600_ reached 0.8. The cells were cooled, induced with 0.4 mm isopropyl β-d-thiogalactopyranoside, and incubated at 16 °C for 16 h. The cells were harvested, resuspended in lysis buffer (25 mm HEPES-NaOH, pH 7.0, 100 mm NaCl, 5% (v/v) glycerol) supplemented with 0.2 mg of DNase (Sigma) and 1× protease inhibitor mixture (Roche Applied Science), and sonicated for 4 min using a probe set at 40% amplitude with pulses of 8 s ON and 10 s OFF. Unbroken cells were removed by centrifugation at 22,000 × *g* for 30 min, and the supernatant was passed through a 0.45-μm filter. The lysate was mixed with 2 ml of nickel-nitrilotriacetic acid resin (Qiagen) and incubated at 4 °C with gentle mixing for 1 h. The resin was packed in a gravity flow column and washed with 20 column volumes of lysis buffer supplemented with 10–50 mm imidazole. Increasing the concentration of the imidazole to 200 mm eluted the TtCinA protein from the resin. Thrombin protease (GE Healthcare) was added to the protein, and the mixture was exchanged with buffer A (25 mm HEPES-NaOH, pH 7.0, 5% (v/v) glycerol) by dialysis for 16 h at 4 °C. The sample was injected into a Resource Q column (GE Healthcare) and eluted using a linear gradient from buffer A to buffer B (25 mm HEPES-NaOH, pH 7.0, 1 m NaCl, 5% (v/v) glycerol). The protein was concentrated using a 10 kDa cut-off centrifugal concentrator (Sartorius) and purified further by using a HiLoad Superdex 75 (16/600) column (GE Healthcare) equilibrated in lysis buffer.

##### Crystallization of TtCinA

The TtCinA protein was concentrated to 13 mg/ml, and sitting drop vapor diffusion experiments were set up using a Mosquito robot (TTP Labtech). 200 nl of protein was mixed with 200 nl of well solution containing 0.2 m Na_2_SO_4_, 0.1 m Bistris propane (pH 6.5), and 20% (w/v) PEG 3350 at 20 °C. Rodlike crystals appeared in 5–7 days. The crystals were washed with well solution and then transferred to the same solution with 25% glycerol (v/v) added and frozen in liquid nitrogen. For ligand binding, crystals were soaked for 5 min in well solution, to which glycerol had been added to 25% (v/v), plus 5 mm MgCl_2_ and 25 mm (final concentrations) of either NMN, ADP-ribose, ATP, or AMP, as appropriate, before freezing in liquid nitrogen.

##### Structure Determination of TtCinA

Details of data collection and refinement statistics are given in Table 1. The data sets were processed by automated pipeline implemented in XIA2 (13) using XDS (14) and AIMLESS (15). The unliganded (“native” in Table 1) TtCinA structure was solved by molecular replacement using PHASER (16) by finding a unique solution (log likelihood gain, −263; translation function *Z* score, −10.5) when two dimers, one each of two different CinA domain structures (Protein Data Bank entries 2A92 and 3KBQ) were placed. The majority of the model was built using AUTOBUILD (17) implemented within PHENIX (18). Model building was completed manually using Coot (19) and refined using phenix.refine (18) and REFMAC (20). The ligand-bound structures of TtCinA were refined using the native structure as a starting model. Clear densities for NCN, ADP-ribose, ATP, and AMP were identified in their respective (*F_o_* − *F_c_*) maps. Apart from docking of the ligands, which was carried out manually in Coot, only minor, manual adjustments to each structure were required. The structures were validated using Molprobity (21) and PROCHECK (22). Coordinates and structure factors were deposited in the Protein Data Bank with the following accession codes: TtCinA apoprotein, 4CT8; NCN complex, 4UOC; ADP-ribose complex, 4UUX; ATP complex, 4CTA; AMP/Mg^2+^ complex, 4UUW.

##### NMN Deamidase and ADPR Assays

To identify the presence of bifunctional enzymatic activities of CinA, an HPLC assay was used where the retention volumes of substrates and products were monitored in a reverse phase column. For the deamidase reaction, 1 ml of buffer (25 mm Tris-HCl, pH 8.0, 100 mm NaCl) containing 100 μg of CinA was mixed with NMN (1 mm) and incubated at 37 °C for 15 min. The reaction was stopped by the addition of 40 μl of trifluoroacetic acid (TFA). The sample was centrifuged at 14,000 × *g* for 10 min to remove the precipitated protein. The supernatant was injected into Synergi C18 250 × 4.6-mm column (Phenomenex) equilibrated with 20 mm ammonium acetate, pH 6.8, and running at 0.5 ml/min. The elution profile of the sample matched with that of the elution profile of the product NCN. NCN eluted (4.9 ml) earlier than the substrate NMN (5.4 ml) (10). A negative control experiment, where no protein was present, was also carried out. Experiments were performed in duplicate. For the ADPR assay, 1 ml of buffer (25 mm Tris-HCl, pH 8.0, 100 mm KCl, 4 mm CoCl_2_) containing 100 μg of CinA was mixed with 1 mm ADP-ribose as substrate. The samples were incubated at 50 °C for 15 min, and the reactions were stopped by the addition of TFA and centrifuged at 14,000 × *g* for 10 min to remove the precipitated protein. Subsequent procedures were identical to those described above for the NMN deamidase assay.

## RESULTS

### 

#### 

##### Enzymatic Activity Measurements

**FIGURE 2.**
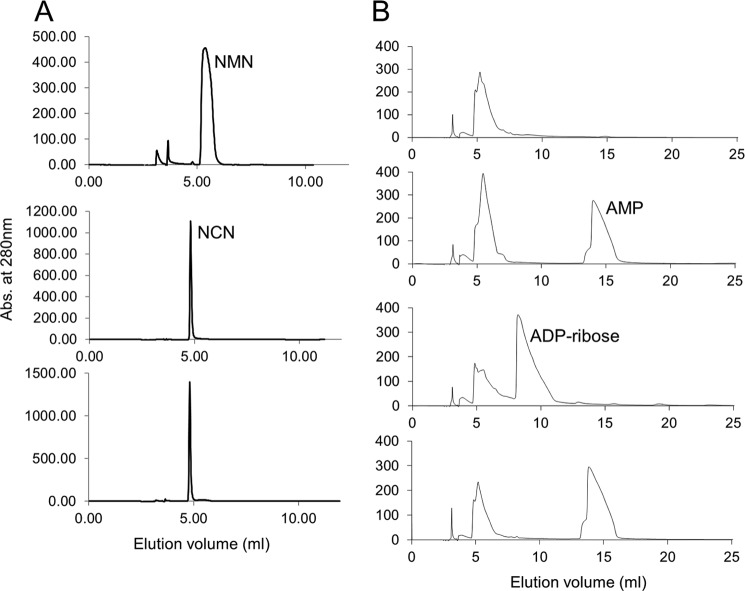
**Assays of NMN deamidase and ADPR activities.** Shown are elution profiles from the reverse phase Synergi C18 250 column. *A*, NMN deamidase activity. *Top*, NMN substrate only (no TtCinA); *middle*, NCN control (no TtCinA); *bottom*, after incubation of NMN with TtCinA. *B*, ADPR activity. *Panels* show the following (in order from the *top*): Co^2+^ buffer-only control, AMP control (no TtCinA), ADP-ribose control (no TtCinA), and ADP-ribose plus TtCinA.

TtCinA was assayed for NMN deamidase and ADPR enzymatic activities by identification of substrate and product peaks eluting from a reverse phase HPLC column (Fig. 2). NMN showed rapid conversion to NCN product in the presence of TtCinA (Fig. 2*A*). Similarly, TtCinA was also capable of conversion of ADP-ribose into AMP (Fig. 2*B*; the other product of the reaction, ribose-5-phosphate, was not detectable in this assay system). The ADPR assay was conducted in the presence of Co^2+^ and K^+^ ions, because these have been identified by Cialabrini *et al.* (11) as conferring optimal activity. The use of a Co^2+^-containing buffer caused an artifactual peak at ∼5.5-ml elution volume; fortunately, this was well separated from the substrate (ADP-ribose; 10 ml) and product (AMP; 15 ml). The observation of these enzymatic activities in TtCinA is consistent with observations made on homologs from other sources (7, 10, 11).

##### Structure Determination, Overall Fold, and Distribution of Domains

**FIGURE 3.**
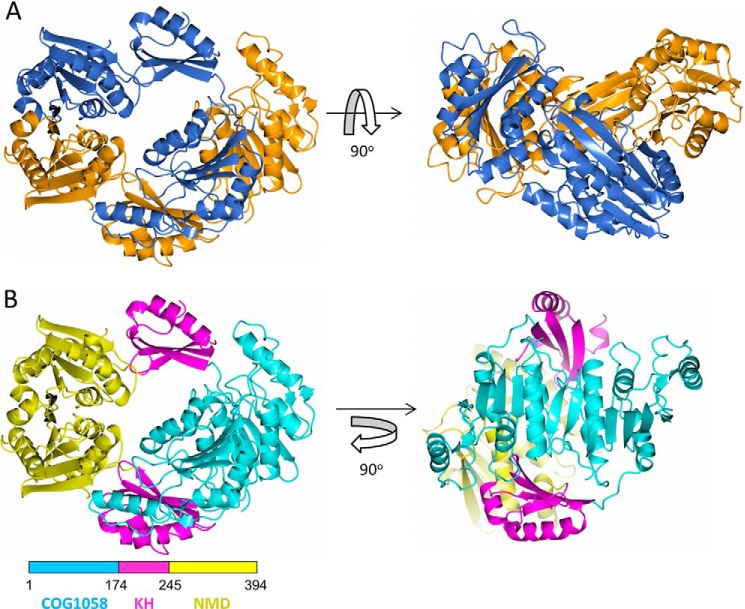
**Overall structure of the CinA dimer.** *A*, two orthogonal views of the CinA dimer. *Orange*, chain A; *blue*, chain B. The apoprotein structure is depicted. *B*, *left-hand view* is the same orientation as the *left-hand panel* of *A*), but each domain is *colored separately*, as shown in the *inset*. The *rotated view* on the *right-hand side* shows the 2-fold axis relating the COG1058 domains (which is not coincident with the 2-fold symmetry axis relating the KH and NMD domains). The figure was prepared using CCP4MG (36).

The crystal structure of TtCinA was solved by molecular replacement using search models corresponding to two of the predicted domains: the NMN deamidase domain (Protein Data Bank code 2A9S) (7) and the COG1058 domain (Protein Data Bank code 3KBQ) (10). As expected, both search models were very similar in structure to the corresponding domains in TtCinA, with 2A9S giving a root mean square deviation of 1.23 Å over the Cα atoms between the two structures, and 3KBQ a root mean square deviation of 1.33 Å. The complete structure forms a dimer, with an overall oval shape (Fig. 3*B*). It was readily apparent that each chain comprises three, not two, domains; the third domain sits between the NMN deamidase (NMD) and COG1058 domains. It forms a KH-type fold, with two α-helices packed against a three-stranded β-sheet (referred to hereafter as the “KH domain”). Most strikingly, the TtCinA dimer is asymmetric; the NMD and KH domains are related by a common 2-fold symmetry axis, which runs horizontally along the main axis of the complex. These two domains superpose between the two chains with a root mean square deviation of 1.1 Å. The COG1058 monomers are also related by a 2-fold symmetry axis (shown from above in Fig. 3*B*), but this is not coincident with the first 2-fold. The KH and NMD domains of chain A form a buried surface area of 815 Å^2^ with the COG1058 domain of chain B, whereas the value for the inverse, *i.e*. the KH/NMD of chain B contacting COG1058 of chain A, is 57 Å^2^ (calculations were performed using PISA (23)). The highest interface area involving a COG1058 domain in crystal symmetry contact is 459 Å^2^; other symmetry contacts are considerably lower. We therefore think that the influence of symmetry contacts on the conformation of the COG1058 domains is relatively minor. Critically, the asymmetry in contacts made by the COG1058 domain results in a difference in the environments of the ADPR active sites on each chain (discussed further below). The formation of a TtCinA dimer in the crystal was corroborated by measurement of molecular mass by size exclusion chromatography, which gave a value of ∼72 kDa, close to the predicted value of 86 kDa expected for a dimer (not shown).

##### NCN Product Complex

**FIGURE 4.**
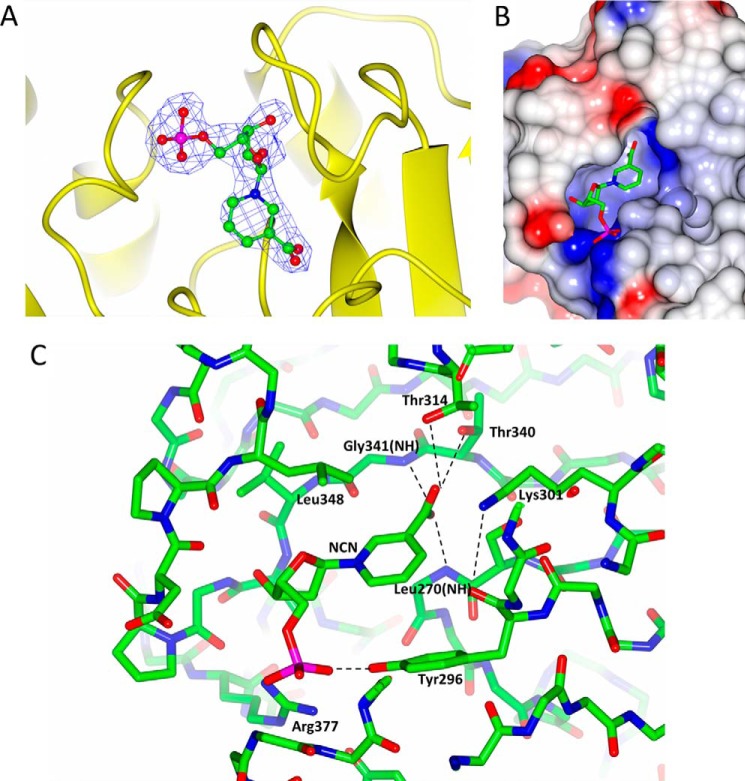
**Binding and recognition of NCN.** *A*, *F_o_* − *F_c_* electron density map in *blue* contoured at 3σ from refinement without NCN, superimposed on a *yellow ribbon plot* of TtCinA and a *ball and stick model* of NCN. *B*, the NCN binding cavity. NCN is shown *superimposed* on an electrostatic surface, which is predominantly positive (*blue*) within the NCN binding site. *C*, TtCinA main chain in the vicinity of the NCN binding site, with selected side chains for key interacting residues *indicated* and *labeled*, along with predicted hydrogen bonds. The figure was prepared using CCP4MG (36).

A data set to 2.46 Å resolution was collected from a TtCinA crystal following a brief soak in the predicted substrate, NMN. Strong electron density for a bound ligand was observed in both NMN deamidase sites (Fig. 4*A*), but, given the chemical similarity between substrate and product, it was not possible to distinguish NMN from the product, NCN. Given the high efficiency with which TtCinA catalyzes this reaction, we built the complex as a product complex, on the basis of the presumption that the enzyme is catalytically active in the crystalline state.

NCN binds in a deep crevice, with the phosphate moiety closest to the surface and the nicotinate portion buried most deeply; consequently, it is this latter part of the ligand that makes the most extensive interactions with residues in the enzyme (Fig. 4*B*). Arg-377 forms a salt bridge to the phosphate, and is likely to make an important energetic contribution to binding (Fig. 4*C*); it is very highly conserved (10). Recognition of phosphate is complemented with hydrogen bonds from the phenolic hydroxyl of Tyr-296 (again, very highly conserved) and the backbone amides of Gly-284 and Ser-286. Two other backbone amides, from Ala-343 and Gly-344, recognize the hydroxyls on the ribose ring. The most critical interactions, however, from the point of view of understanding catalysis, are with the nicotinate ring. The most important residues in this respect are Thr-314, Thr-340, and Lys-301 (Fig. 4*C*); Lys-301 is very highly conserved, whereas Thr-340 is Ser in some sequences (although Thr in most), and Thr-314 is Ala in many sequences. We propose a role for Lys-301 as a general base in the reaction, working in concert with Thr-340 (see below).

##### ADPR

**FIGURE 5.**
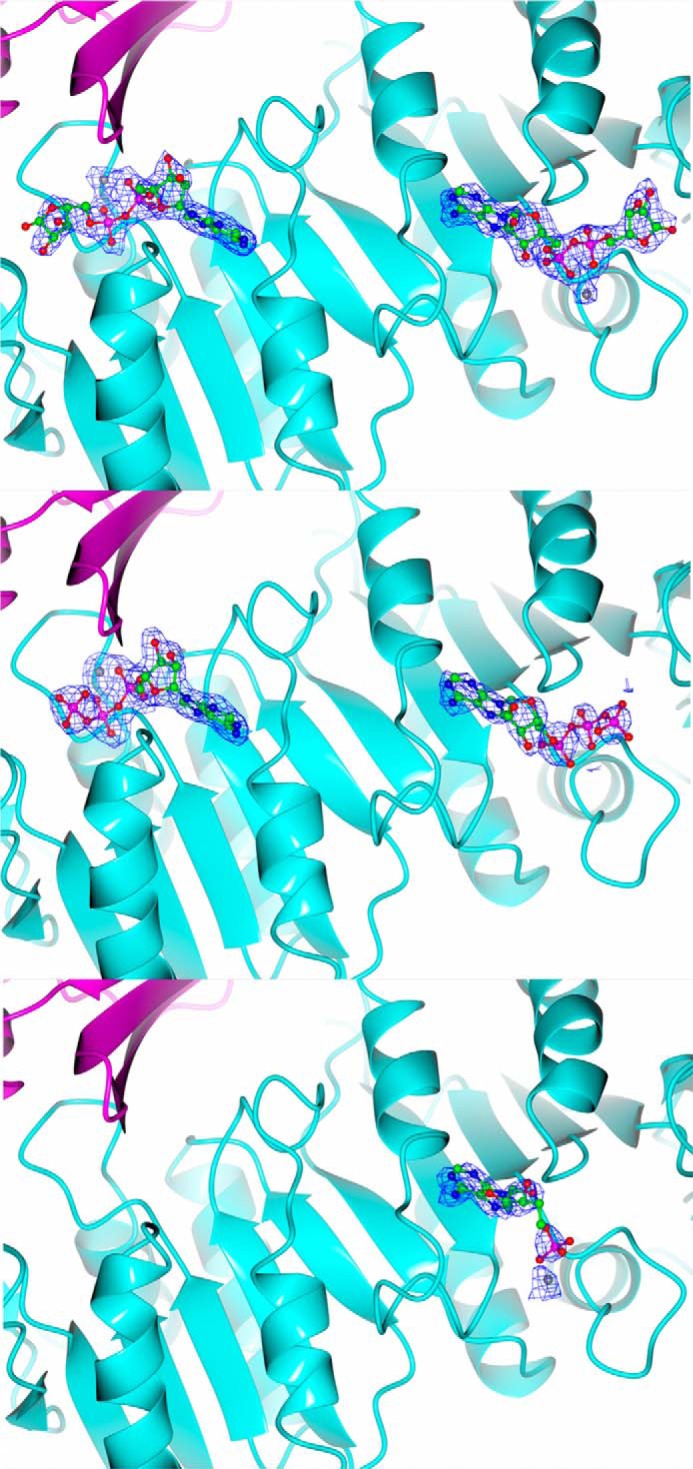
**Ligand binding to the ADP-ribose pyrophosphatase sites.** *F_o_* − *F_c_* density maps, in *blue*, were calculated from refinement without bound ligand, to remove phase bias. In each case, the ligand is shown *superimposed* on the relevant density map, contoured at 3σ, with the ligand and a *ribbon plot* of TtCinA. Colors of the COG1058 and KH domains are as used in Fig. 3. Complexes are, from *top* to *bottom*, ADP-ribose, ATP, and AMP. In each case, the open site is on the *right-hand side* of the image. Some domains have been removed for clarity. The figure was prepared using CCP4MG (36).

Data were collected from three different complexes, following soaking of TtCinA crystals with ADP-ribose, ATP, and AMP/ribose 5-phosphate (Table 1). Both the ADP-ribose and ATP data sets produced clear difference density in both catalytic sites (Fig. 5, *top* and *central panels*). There was no indication of hydrolysis of ADP-ribose or ATP; this may be due to a slowing of catalysis in the crystalline state. Density was considerably weaker for the AMP data; however, it was possible to model in an AMP molecule in one of the two active sites (Fig. 5, *bottom*), and density for a bound Mg^2+^ ion was observed in both sites. A separate experiment in which TtCinA crystals were soaked with ribose 5-phosphate alone produced no clear density for the ligand in either binding site (not shown).

**TABLE 1:** **Data collection and refinement statistics**

	Native	NCN	AMP/Mg^2+^	ATP/Mg^2+^	Ribose-ADP/Mg^2+^
**Data collection**					
Space group	P 2_1_2_1_2_1_	P 2_1_2_1_2_1_	P 2_1_2_1_2_1_	P 2_1_2_1_2_1_	P 2_1_2_1_2_1_
Unit cell parameters (Å)	72.97,93.55, 132.69	72.47,93.30, 133.77	73.28,93.71, 132.82	74.59,93.77, 131.79	73.39, 94.03, 132.38
X-ray source and wavelength (Å)	DLS*^a^* IO4–1 (0.92)	DLS I04 (0.98)	DLS I03 (0.90)	DLS I03 (0.90)	DLS I03 (0.90)
Resolution range (Å)	54–2.16 (2.22–2.16)*^b^*	93–2.46 (2.52–2.46)	53–1.98 (2.03–1.98)	66–2.21 (2.27–2.21)	29–1.99 (2.04–1.99)
Multiplicity	4.9 (4.6)	3.5 (3.4)	3.4 (3.4)	3.6 (3.7)	4.2 (4.2)
Significance (〈I〉/S.D.)	12.1 (2.2)	7.9 (2.0)	12.4 (2.3)	11.2 (2.0)	17.0 (2.5)
No. of unique reflections	49,274	31,049	63,973	46,732	62,924
Completeness (%)	99.7 (99.4)	93.4 (95.9)	99.5 (99.6)	99.4 (99.2)	99.3 (99.0)
*R*_merge_ (%)*^c^*	9.2 (59.5)	12.5 (69.7)	5.4 (46.5)	9.8 (57.2)	5.7 (56.0)

**Refinement statistics**					
*R*_cryst_	18.8	18.9	20.0	22.0	17.6
*R*_free_	24.1	25.0	23.6	27.4	21.2
Non-hydrogen atoms					
All	6,309	6,237	6,309	6,300	6,587
Water	318	232	318	132	463
Mean overall *B* (Å^2^)	31.8	33.3	32.3	36.1	31.4
Root mean square deviation from ideal values					
Bond distance (Å)	0.016	0.014	0.019	0.017	0.021
Bond angle (degrees)	1.5	1.8	2.0	1.8	2.0

*^a^* Diamond Light Source.

*^b^* Values in parentheses refer to the outer resolution shell.

*^c^ R*_merge_ = Σ*_hkl_*Σ_sym_|*I* − 〉*I*〉|/Σ*_hkl_I*.

**FIGURE 6.**
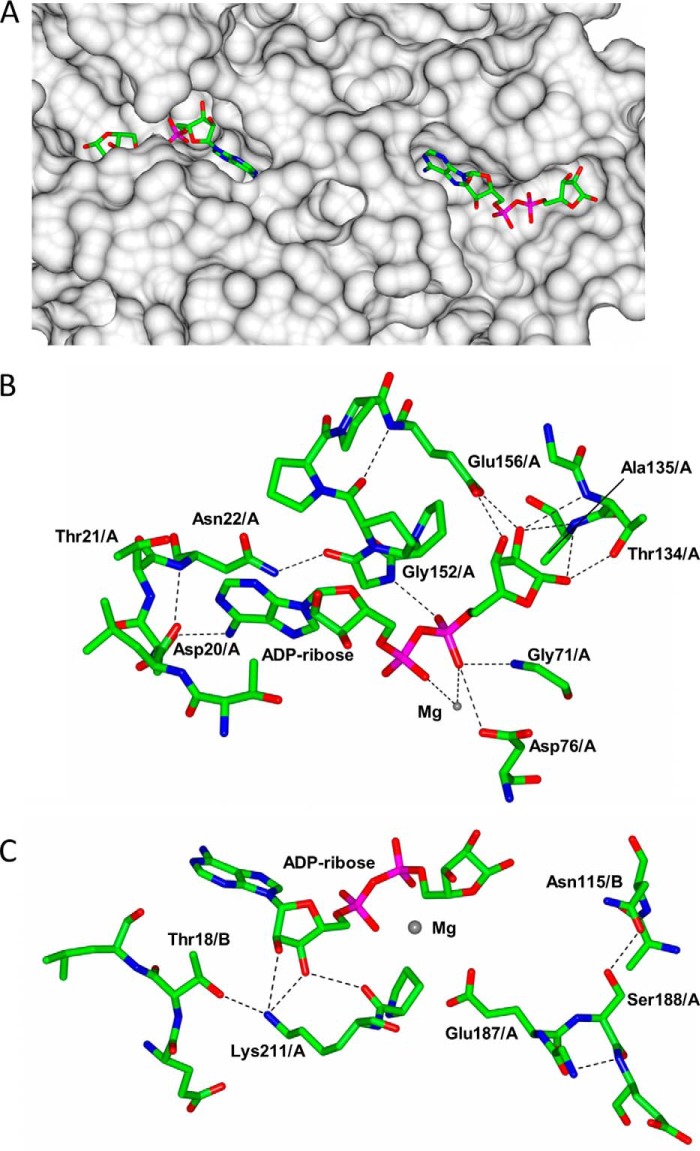
**Binding and recognition of ADP-ribose/Mg^2+^.** *A*, molecular surface, computed using CCP4MG (36), with ADP-ribose superimposed; the *left-hand side* shows the “closed” site, and the *right-hand side* shows the “open” site. Some domains were removed from the surface calculation for clarity. *B*, detail of the open ADP-ribose binding site; selected interacting residues from chain A are indicated. *C*, interactions of the chain A KH-like domain with ADP-ribose and selected residues from chain B COG1058 from the “closed” ATP binding site.

The asymmetric nature of the TtCinA dimer has important ramifications for the ADP-ribose binding sites; for chain A, the site forms a more open structure, whereas for chain B, it is almost sealed by close contact with the KH-like domain from chain A. The relative access to the two sites is illustrated in Fig. 6*A*; in the closed form, ADP-ribose is bound in a deep cleft, with the terminal ribose ring at one end and the adenine ring at the other. The open ADP-ribose binding site, by contrast, is more readily accessible to bulk solvent. Examination of the recognition of ADP-ribose in the open binding site (chain A) showed a hydrogen bond from Asp-20 to the adenine ring (Fig. 6*B*). A strong peak in the electron density map, inferred to be from Mg^2+^, was identified between the α and β phosphates. Such a location would be consistent with pyrophosphatase activity. Asp-76 binds the Mg^2+^ ion and is absolutely conserved in CinA sequence alignments. Glu-156 appears to play an important part in recognition of the terminal ribose ring; it forms two hydrogen bonds to the 2′- and 3′-hydroxyl groups in the ribose ring and is also absolutely conserved in sequence alignments. Recognition of ADP-ribose in the closed site is very similar to that in the open but with the important exception that there are additional points of contact between the ligand and TtCinA from residues in the KH domain of chain A. The side chain of Lys-211 and the carbonyl oxygen of Pro-210 form hydrogen bonds to the 2′- and 3′-hydroxyl groups in the ribose moiety in the ADP part of the molecule (Fig. 6*C*). In addition, the binding of the Mg^2+^ ion appears to be stabilized by Glu-187, which extends from the end of an α-helix in the KH domain into the ADP-ribose binding site.

**FIGURE 7.**
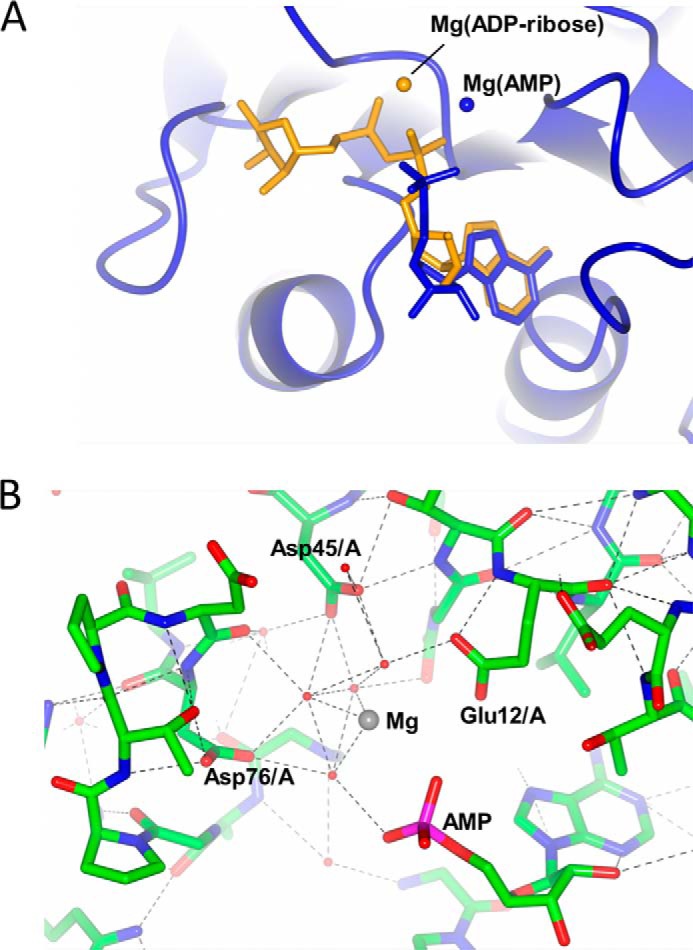
**Binding and recognition of AMP/Mg^2+^.** *A*, comparison of AMP and ADP-ribose binding in the “open” site. ADP-ribose is shown in *orange*; the AMP complex, ligand and AMP protein, is shown in *blue*. The two locations of the Mg^2+^ ion in each complex are indicated. *B*, detail of Mg^2+^ binding in the AMP complex; key interacting residues are indicated.

ATP binds in a very similar conformation to ADP-ribose, with the γ-phosphate occupying a location similar to that of the terminal ribose ring. Mg^2+^ binds between the α- and β-phosphates, as in the ADP-ribose complex. ATP therefore appears to bind in a manner similar to ADP-ribose. By contrast, there are subtle but important differences in the manner of AMP binding. First, density for AMP was only sufficiently strong in the open site to allow stable refinement of the ligand. Second, although the position of the adenine ring in AMP is similar to that in the ADP-ribose and ATP complexes, the α-phosphate is displaced outward (Fig. 7*A*). Third, the location of the Mg^2+^ ion is also different; it is displaced by 3.0 Å from its position in the substrate complex. A network of water molecules are bound around the ion, and it is flanked by the α-phosphate on AMP on one side and by Asp-76, Asp-45, and Glu-12 on the other (Fig. 7*B*). These latter two residues are much closer to the Mg^2+^ ion in this product complex, and both are absolutely conserved in CinA sequences. These observations suggest that movement of the Mg^2+^ ion occurs as part of the catalytic cycle.

The observation that AMP appears to bind preferentially to the open site suggests that product release occurs from this location. Conversely, the closed site is more likely to correspond to the catalytically competent state; critically, the close approach of the KH domain from chain A, with Glu-187, would promote the binding of Mg^2+^ and hence polarize the P=O bond for hydrolysis. These inferences have allowed us to construct a model that relates the movement of the COG1058 domains to the ADPR catalytic cycle.

## DISCUSSION

Earlier studies on CinA have identified that, in about 40% of sequences (10), the NMN deamidase domain is fused to a COG1058 domain, which has been associated with ADPR activity (11). The structural relationship of these domains in the bifunctional enzyme has not been studied to date, however, and current structures of representatives of these domains do not have ligands bound. This has limited the interpretation and understanding of the mechanisms of both reactions.

Enzymological studies on other carboxylic acid amide hydrolysis reactions are potentially informative in understanding the mechanism of NMN deamidase. For example, l-asparaginase II (EC 3.5.1.1) forms l-aspartate and NH_3_ from l-asparagine and water, and the enzyme has been well studied from prokaryotic and eukaryotic sources (24). Parallels have been drawn between the general mechanism for l-asparaginase and serine proteases, the latter classically requiring a nucleophile residue (Ser), a base (His), and an acidic residue (Asp) connected by a hydrogen bonding network (25). The *Escherichia coli* enzyme asparaginase II contains a similar set of residues: Thr-12, which can act as a nucleophile, and, on the opposite side of the substrate, Lys-162 and Asp-90 (24, 26). Earlier mechanisms for l-asparaginase II proposed a reaction that involved formation of an acyl-enzyme intermediate from the attacking nucleophile, with a concomitant release of NH_3_ (24). Recently, some doubt has been cast on this mechanism, specifically the presence of an acyl-enzyme intermediate in the reaction pathway (27). The T12A mutant retains some activity, albeit only 0.04% of wild type (28). Using a QM/QM computational approach, Gesto *et al.* (27) put forward an alternative mechanism that involves direct nucleophilic attack by a water molecule, followed by collapse of the resulting tetrahedral intermediate and elimination of NH_3_. This has the advantage that it avoids formation of an acyl-enzyme intermediate but is apparently consistent with other data on the enzyme mechanism.

**FIGURE 8.**
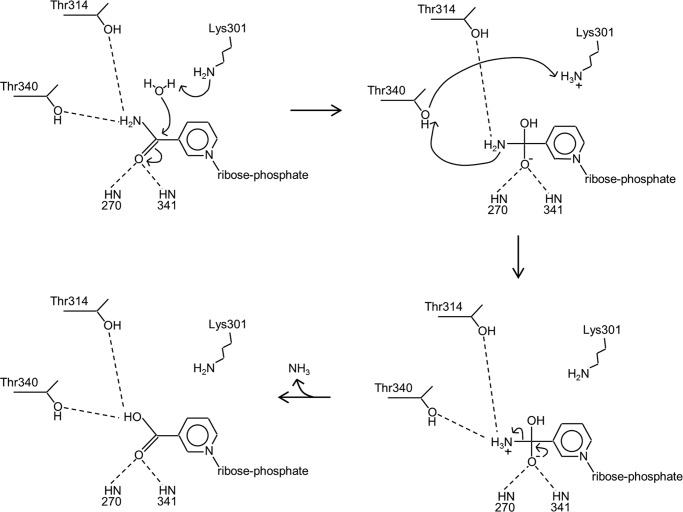
**Proposed mechanism for NMN deamidase.**

A proposed mechanism for NMN deamidase, adapted from that advocated by Gesto *et al.* (27) for asparaginase II, is shown in Fig. 8. Reaction is initiated by nucleophilic attack by a water molecule, promoted by Lys-301, which acts as a general base. Calculations of p*K_a_* values were made with a finite difference Poisson-Boltzmann method (29), for which protein and solvent relative dielectric values of 4 and 78.4 were used, and ionic strength was set to 0.15 m. Based on the structure of the NCN complex but with substrate, NMN, a p*K_a_* value for Lys-301 of around 8 was computed. This value could be adequate for Lys-301 to act as a general base in the reaction, considering that the loss of water solvation alone is sufficient to reduce the p*K_a_* of Lys-301 to neutral pH but that compensating favorable partial charge interactions (which will depend on conformational details) bring the p*K_a_* up to the computed value. Examination of the pH dependence of the reaction catalyzed by PncC from the alkaliphilic bacterium *Oceanobacillus iheyensis* showed little variation from pH 10 down to 7 but with a substantial drop at acid pH (10). The resulting tetrahedral intermediate then rearranges to eliminate NH_3_, with the participation of Thr-340 and Lys-301. We note that Thr-340 is very well conserved in sequence alignments, with only some species having a Ser at this position. Thr-314, on the other hand, is frequently found as Ala; we therefore think that it is unlikely to play a fundamental role in the mechanism, although it may assist in the initial nucleophilic attack step. Lys-301 is absolutely conserved. We note also the important role played by the back bone amide NH groups of residues 270 and 341 in stabilizing the anionic reaction intermediate. For our mechanism to be plausible, the OH group of Thr-340 and NH_2_ group of Lys-301 would need to be close in space: molecular modeling of the Thr-340 and Lys-301 side chains established that they could be placed less than 2.4 Å apart without movement of the main chain atoms. We therefore propose this mechanism as most consistent with the structural data we have presented and with current knowledge of the reaction mechanisms of similar enzymes.

**FIGURE 9.**
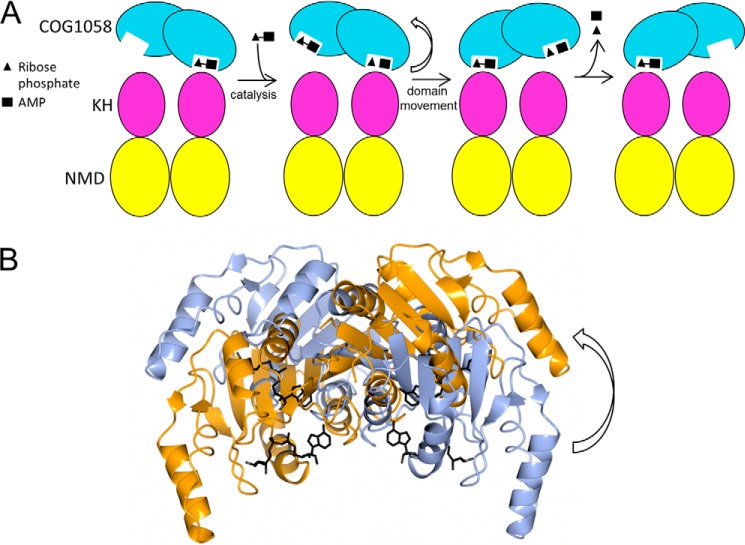
**Proposed domain movement mechanism for ADPR.** *A*, *outline scheme* for linkage of domain movement to catalysis. Domain *colors* are as for Fig. 3. *B*, movement of COG1058 domains; the two alternate conformations of the COG1058 domains are shown for chains A and B, with the NMD and KH-type domains fixed (but not shown). The view is orientated approximately along the rotation axis, so that transition from one orientation to the other requires a rocking motion of ∼51°. The structure shown is the ADP-ribose-bound form (Protein Data Bank entry 4UUX).

A major feature of the TtCinA structure is the asymmetry in the dimer, which is caused by contact between a KH-type domain on the opposite chain and the bound ADP-ribose. This has the effect of closing the active site, allowing additional recognition of ADP-ribose by residues from the KH-type domain (Fig. 6*C*). An analysis of the sequences of the KH-type domains from other CinA proteins provides some additional evidence in support of the importance of this interaction. Glu-187 is absolutely conserved and is part of a highly conserved pentapeptide (GIGES), which forms a loop between an α-helix and a β-strand in the KH domain. AMP appears to bind preferentially to the open site, and electron density was generally weaker than for ADP-ribose and ATP. These observations suggest a general scheme for ADPR catalysis, which is shown in Fig. 9. ADP-ribose is bound in the closed, catalytically competent state; it then undergoes hydrolysis to give ribose phosphate and AMP. Binding of a second ADP-ribose can occur to the open site (which, we reason, is more likely to be receptive to substrate binding). We then suggest that the COG1058 domains rotate relative to the KH and NMD domains, swapping the closed and open sites. In this form, the AMP and ribose phosphate products can leave, vacating a binding site for another ADP-ribose molecule. The movement of the bound Mg^2+^ ion may play a role in reducing binding affinity for the AMP product, helping it to leave (Fig. 7). Such a scheme would explain the need for an additional KH domain in the bifunctional enzyme and provide a rationale for the asymmetric dimer structure that we observe. It may also explain the relatively low rate of hydrolysis that we observe with the ADPR reaction, if it is associated with large domain movements of the KH-type domain.

Homodimers with asymmetrically arranged domains are relatively rare; indeed, departures from perfect symmetry are generally confined to more local perturbations, such as register displacements between adjacent β-strands or axial staggers between α-helices (30). Goodsell and Olsen invoked an evolutionary argument to explain this observation; the domain-domain interface in an asymmetric complex must be optimized for both molecular environments (*e.g.* a solvent-exposed hydrophilic surface) and a more hydrophobic-type surface (*e.g.* associated with a protein-protein interface) (31). In the case of TtCinA, this would correspond to the recognition of ADP-ribose and residues in the COG1058 domain by the KH domain, as shown in Fig. 6*C*. Here we would argue that the constraints imposed by the requirement for catalysis overcome the evolutionary pressure that would otherwise force the dimer to form a conventional homodimer with C2 symmetry.

Apart from TtCinA, *T. thermophilus* also harbors two other enzymes with ADPR activity (32). They form part of a larger group of Nudix proteins, which catalyze the hydrolysis of nucleoside diphosphates (33, 34). Wakamatsu *et al.* advocated the division of ADPRs into two general classes; class I has a wider specificity, whereas class II is more specific for ADP-ribose (32). In *T. thermophilus*, Ndx2 is capable of hydrolyzing both ADP-ribose and FAD (hence class I (32)), whereas Ndx4 does not hydrolyze FAD and is more specific for ADP-ribose (class II (35)). The structures of both enzymes are similar, although they only share 25% sequence identity. However, there is little obvious structural relationship between Ndx4/Ndx2 on the one hand and the COG1058 domain on the other; they both adopt α/β folds but form dimers in different ways, and the binding of substrates is to different, although overlapping, parts of the structure. Consequently, there is no similarity between the key residues involved in substrate recognition and catalysis in Ndx4/Ndx2 and TtCinA. The reason why a particular organism requires multiple ADPRs is unclear, although it is a common phenomenon. In the case of TtCinA, it is tempting to speculate that, because both enzymatic activities are linked to competence, they may form part of a regulated physiological response that accompanies induction of the competent state. One aspect of future work could examine the relationship between TtCinA expression and other components of the competence pathway.

## Footnotes

2 The abbreviations used are:

NMN: nicotinamide mononucleotide
NCN: nicotinate mononucleotide
NMD: nicotinamide mononucleotide deamidase
ADPR: ADP-ribose pyrophosphatase
TtCinA: *T. thermophilus* CinA
Bistris propane: 1,3-bis[tris(hydroxymethyl)methylamino]propane.
